# Fast PET Scan Tumor Segmentation Using Superpixels, Principal Component Analysis and K-Means Clustering

**DOI:** 10.3390/mps1010007

**Published:** 2018-01-19

**Authors:** Yeman Brhane Hagos, Vu Hoang Minh, Saed Khawaldeh, Usama Pervaiz, Tajwar Abrar Aleef

**Affiliations:** 1 Erasmus+ Joint Master Program in Medical Imaging and Applications, University of Burgundy, 21000 Dijon, France; minhmanutd@gmail.com (V.H.M.); khawaldeh.saed@gmail.com (S.K.); 12beeupervaiz@seecs.edu.pk (U.P.); tajwar_aleef@etu.u-bourgogne.fr (T.A.A.); 2 Erasmus+ Joint Master Program in Medical Imaging and Applications, UNICLAM, 03043 Cassino FR, Italy; 3 Erasmus+ Joint Master Program in Medical Imaging and Applications, University of Girona, 17004 Girona, Spain; 4 Department of Electrical Engineering and Automation, Aalto University, 02150 Espoo, Finland

**Keywords:** k-means, positron emission tomography, principal component analysis, segmentation, superpixels

## Abstract

Positron Emission Tomography scan images are extensively used in radiotherapy planning, clinical diagnosis, assessment of growth and treatment of a tumor. These all rely on fidelity and speed of detection and delineation algorithm. Despite intensive research, segmentation has remained a challenging problem due to the diverse image content, resolution, shape, and noise. This paper presents a fast positron emission tomography tumor segmentation method using superpixels. Principal component analysis is applied on the superpixels and their average value. The distance vector of each superpixel from the average is computed in the principal components coordinate system. Finally, k-means clustering is applied on the distance vector to recognize tumor and non-tumor superpixels. The proposed approach is implemented in MATLAB 2016A, and promising accuracy with execution time of 2.35 ± 0.26 s is achieved. Fast execution time is achieved since the number of superpixels, and the size of distance vector on which clustering was done are low compared to the number of pixels in the image.

## 1. Introduction

Positron emission tomography is a non-invasive nuclear medicine afunctional imaging method that images the distribution of biologically targeted radiotracers with high sensitivity. Positron emission tomography (PET) imaging provides detailed quantitative information about the number of diseases and is often used to evaluate cancer with segmentation as a principal role. Image contrast enhancement is an essential pre-processing stage in image segmentation [1]. For several years, great effort has been devoted to the study of image enhancement techniques; wavelet-contourlet transform [2], iterative denoising and partial volume correction [3], iterative deconvolution [4] have been among them.

Segmentation can be thought of as two consecutive processes: recognition and delineation. Recognition is determining where the targeted object is in the image, while the second process is defining the spatial extent of the recognized region [5]. It has been demonstrated that manual segmentation is time-consuming, labor intensive, operator dependent, subjective, and these make it less precise and reproducible [6,7]. In the recognition process, regions of high uptake of tracer are identified either manually or automatically [8].

Although the number of PET image segmentation publications has always been lower than both computerized tomography (CT) and magnetic resonance imaging (MRI) [6], there have been some publications; graph cut and locally connected conditional random field via energy minimization [9], binary and Gaussian filtering regularized level set method with capability of detecting weak tumor boundary [10]. In addition, k-means and fuzzy c-means clustering-based segmentation has been developed [11]; however, clustering was applied to image pixels directly and this is computationally demanding for large-sized images and/or, as the number of clusters is higher.

Principal component analysis (PCA)-based evaluation of internal statistics of image patches gives tremendous insight to recognizing patterns in an image [12], which is applied to detect salient objects in a natural image.

This paper presents the implementation of an unsupervised automatic PET image segmentation system to detect a tumor. Section 2 presents the mathematical formulation and implementation of proposed approach which contains, contrast enhancement superpixels, and PCA followed by k-means clustering to recognize the cancer superpixels. Section 3 is devoted to discussion and evaluation of the simulation results. Finally, Section 4 concludes the paper.

## 2. Implementation

**Figure 1:**
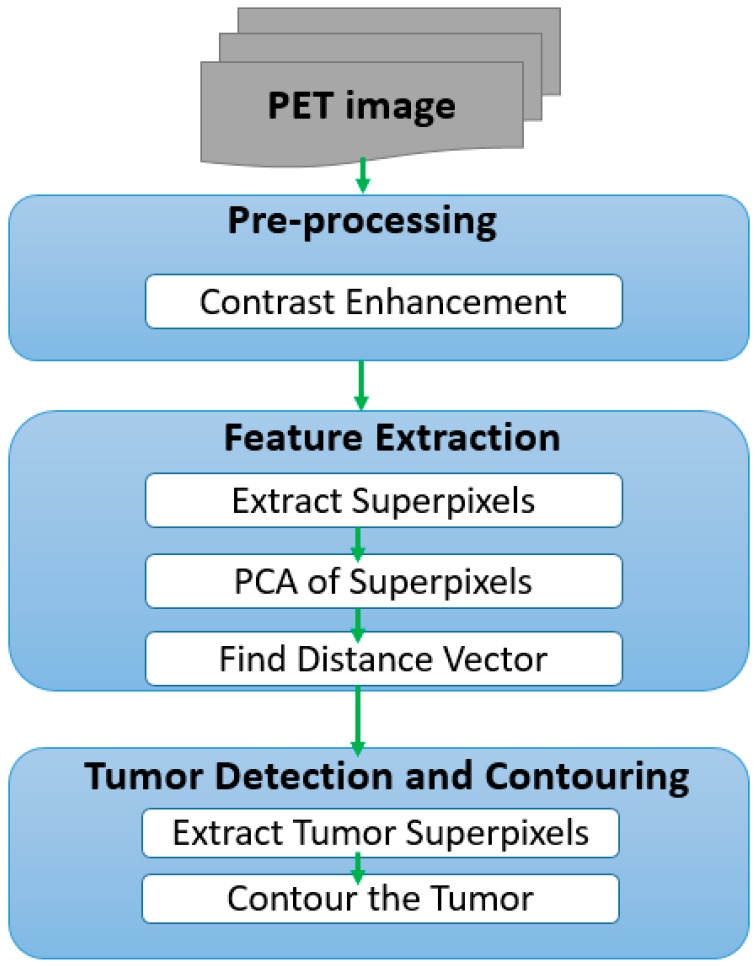
Implementation overview. PET: Positron emission tomography; PCA: principal component analysis.

The workflow of the proposed approach is divided into three stages: pre-processing, feature extraction and clustering, and segmentation; where the second step can be divided into three sub-steps, and the third step into two as shown in Figure 1.

### 2.1. Pre-Processing

Image enhancement is a subjective process and its goal is to make the image suitable for the next steps. In this paper, we have applied piecewise contrast enhancement. Upon extensive analysis of different images, we have found that piecewise linear stretching shown in Equation (1), improves the overall performance of our implementation. However, to apply this enhancement to other modality images, it might be needed to tune its parameter.

(1) $$I_{enh}=\{\begin{array}{lll} \;\;I & , & if\;I\geq \;110\\ \frac{55}{145}\left(I-110\right)+200 & , & Otherwise \end{array}$$

where *I* is input image and *I_enh_* is contrast enhanced image.

### 2.2. Feature Extraction

Feature extraction is a process of simplifying the content of a large set of data to describe it efficiently for the purpose of facilitating further processing (speed), storage requirement, and dimensionality reduction. In this paper, features are extracted using superpixels and PCA.

A superpixel is a group of pixels which are in close proximity and sharing similar intensity. The simple linear iterative clustering (SLIC) algorithm [13] is applied due to its fast computational time as stated in [14,15]. The size of a superpixel is the number of pixels belonging to that superpixel. The size of original superpixels extracted from SLIC is different as there is a large homogenous region in some part of the image resulting in large superpixels, while there might also be a small number of pixels near each other with similar pixel value in some region of the image. Generally, superpixels in the background and non-tumor part of the image will have a large number of pixels.

However, we need the size of superpixels to be the same to perform PCA. This problem is solved as follows:

(1) We computed the average size of the superpixel as shown in Equation (2).
  (2) $$M\;=\;\frac{1}{N}\overset{N}{\underset{i=1}{\sum }}n_{i}$$
  where *N* is the number of superpixels, and *n_i_* is the number of pixels in *i*th superpixel. Then, *M* is an average number of pixels per superpixel.
(2) Then, the size of each superpixel is made same as that of the average one by padding some pixel value to the smaller size superpixel and removing some intensity value from the large size superpixels. Instead of appending random intensity values to smaller sized superpixels, we pad by repeating the last pixels value of the superpixel itself. Finally, the superpixel matrix is generated as shown in Equation (3)
  (3) $$S=\;\left[\begin{array}{cc} \begin{array}{ccc} x_{11} & x_{12} & x_{13}\\ x_{21} & x_{22} & x_{23}\\ . & . & . \end{array} & \begin{array}{ccc} .\;\;. & . & x_{1N}\\ .\;\;.\; & . & x_{2N}\\ .\;\;. & . & . \end{array}\\ \begin{array}{ccc} . & . & .\\ . & . & .\\ x_{M1} & x_{M2} & x_{M3} \end{array} & \begin{array}{ccc} .\;\;. & . & .\\ .\;\;. & . & .\\ .\;\;. & . & x_{MN} \end{array} \end{array}\right]$$
  where each column represents a superpixel pixel, *M* is in Equation (2) and *N* is the number of superpixels.

As the goal is to detect pixel that belong to the cancer or tumor, and in PET images, pixels that belongs to tumor have distinct intensity due to high uptake of radioactive tracer; so we need a method that analyses the internal statistics and makes easy to differentiate the cancer superpixels. Principal component analysis is one of the novel methods to study internal statistics of data. In addition to that, PCA reduces the dimensional space of the data [16]. In our implementation, PCA of superpixels is done as follows:

(1) Compute average superpixel.
  (4) $$S_{a}=\;\frac{1}{N}\overset{N}{\underset{i=1}{\sum }}S_{i}$$
  where *S_i_* is the *i*th superpixel and *S_a_* average superpixel.
(2) Determine the covariance of superpixels (*C_s_*)
  (5) $$C_{s}=\;\frac{1}{N+1}\left(Y\;-\;Y_{a}{}^{t}\right){\left(Y\;-\;Y_{a}{}^{t}\right)}^{T}$$
  where *Y* is the superpixel matrix after average superpixel padding and *Y_a_^t^* is the mean of transpose of *Y*.
(3) Calculate the eigensuperpixels (eigenvectors) and eigenvalues of the covariance matrix
  (6) $$C_{s}=\;P\Sigma P^{T}$$
  where *P* is matrix with eigensuperpixels as column and Σ is diagonal matrix of eigenvalues, λ_1_, λ_2_, …, λ_N_, where, λ_1_ ≥ λ_2_ ≥ λ_3_…≥ λ_N_.
  The magnitude of eigenvalue shows the variance of the data in the direction of its corresponding eigensuperpixel. For *N* superpixels in Equation (3) above, total variance of intensities of the M- dimensional superpixels can be computed in terms of eigenvalues from Equation (7).
  (7) $$V=\overset{N}{\underset{k=1}{\sum }}\lambda_{k}$$
  where *V* is the total variance.
(4) Project the superpixels onto eigensuperpixels that contain most of variance of the data. In Equation (6), the number of principal components is same as the number of superpixels. As stated in [17], the eigenvectors or principal components that contain at least 95% of the variance of superpixels can represent the whole image with confidence and this is computed as shown in Equation (8). It reduces the dimensional space, as most of the information is contained in the first two or three largest eigenvalues.
  (8) $$\overset{K}{\underset{k=1}{\sum }}\lambda_{k}\;\leq 0.95$$
  In our test images, 95% of the variance of superpixels was in the top two (*K* = 2) principal components, with most of them reducing M-dimensional superpixels to a 2D point. Once the dominant vectors are found, for feature extraction, the superpixel matrix is projected onto these vectors using Equation (9).
  (9) $$Y_{proj}=P_{K}^{T}Y$$
  where *P_k_* is eigenvectors matrix that contains at least 95% of the variation in the image and *P_proj_* is projection of superpixel matrix to *P_k_*.
(5) Calculate the distance of each superpixel to average superpixel. Computing distance should consider the distribution of superpixels in the principal component coordinate system [12]. To incorporate this concept, we computed the distance along the principal components. Mathematically, this will be computing *L*_1_ norm distance in the principal components coordinate system as shown in Equation (10) below.
  (10) $$D\left(S_{i}\right)=||S\prime_{i}|{|}_{1}$$
  where *S′_i_* is coordinate of *S_i_* relative to *S_a_* in the principal component coordinate system, and *D* is *L*_1_ norm distance.

### 2.3. Tumor Detection and Contouring

Currently, there are a variety of PET segmentation methods. The most commonly used method is fuzzy locally adaptive Bayesian (FLAB), classification/clustering, and a mixture of them. As stated in [6] there is a growing need for clustering-based methods as they have the capability of detecting tumors with a complex shape in heterogeneous PET images. In our work, after the distance vector is calculated in the principal components coordinate system, k-means clustering is applied. k-means is an algorithm that clusters a set of data based on a distance measure. This clusters superpixels as tumor and non-tumor, which is a binary classification using a minimization problem as shown in Equation (11). Then, morphological operations (erosion and dilation) are then applied to delineate the spatial scope of the tumor.

(11) $$\underset{C}{\mathrm{argmin}}\overset{2}{\underset{i=1}{\sum }}\underset{X\;\in \;C_{i}}{\sum }D=\underset{C}{\mathrm{argmin}}\overset{2}{\underset{i=1}{\sum }}\underset{X\;\in \;C_{i}}{\sum }||X-\mu_{i}||{}_{2}^{2}$$

where *c_i_* is the set of points that belong to cluster *i*, *µ_i_* is center of *i*th cluster, *X* is distance vector extracted above and *D* is square of the Euclidean distance.

## 3. Result and Discussion

**Figure 2:**
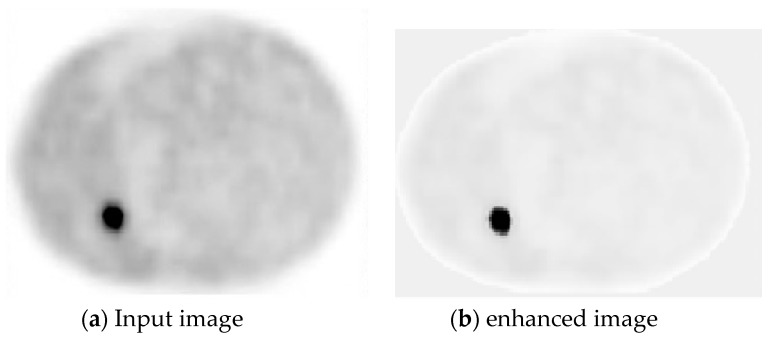
Image before and after enhancement.

Figure 2a,b below shows the input image and enhanced image respectively. It can be clearly seen that contrast between tumor and non-tumor region of the image is enhanced.

**Figure 3:**
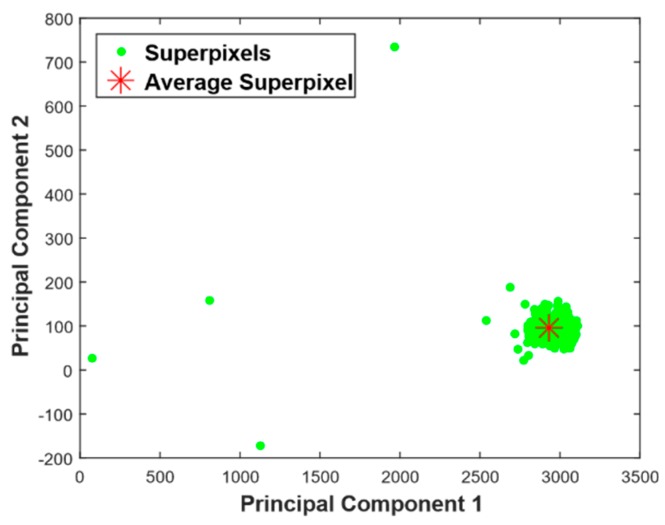
Scatter plot of projection of superpixels of the enhanced image onto the principal components classification is small.

For the input image in the figure, 692 superpixels were extracted and more than 95% of the variance of superpixels was contained in the top two eigensuperpixels. The scatter plot after the projection onto the top two dominant eigensuperpixels is plotted in Figure 3. In the figure, the principal component 1 is an eigensuperpixel with the highest eigenvalue or the component that constitutes the highest variance of superpixel intensities, while principal component 2 has the second highest eigenvalue among all eigensuperpixels. In the plot, high-dimensional superpixels are represented by a two-dimensional vector. From the scatter plot, it is evident that most of the superpixels are concentrated around the average superpixel (red asterik) as most parts of the image have similar pixel intensity distribution, while the superpixels that correspond to tumor are situated far from it. There are 693 points (692 superpixels + average superpixel) in the scatter plot. 

**Figure 4:**
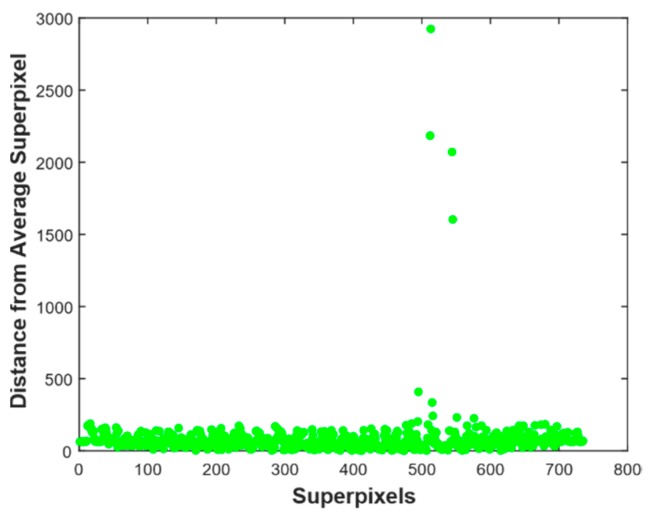
Distance of superpixels from average superpixel.

**Figure 5:**
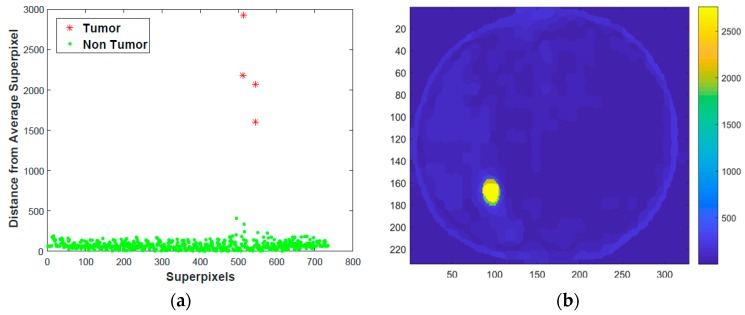
Superpixel k-means clustering and Heat map plot. (**a**) Superpixel k-means clustering; (**b**) heat map plot.

Figure 4 illustrates *L*_1_ norm distance of superpixels along principal components coordinate system from average superpixel. It has superpixel index as horizontal axis and distance as a vertical axis. For the input image in Figure 2a is 233 pixels by 328 pixels, the distance is 692 dimension vector. If distance was computed from all pixels to average pixels intensity the result will be a 76424-dimensional vector, which needs larger memory and high computation time. As depicted in Figure 5, most of the superpixels are within *L*_1_ distance of 500, while 4 superpixels have a distance greater than 1500.

Figure 5a shows k-means clustering of distance vector in Figure 4. Superpixels with low distance are normal or non-tumor (represented by green dots). Superpixels extracted from tumor region (red asterisk) are far from average superpixel. In addition to that, heatmap of distance of superpixels from the average in the image space are shown in Figure 5b. The tumor area is shown in yellow color which is more distinguishable from the other superpixels and has large distance as depicted in the color bar. The internal statistics of the tumor superpixels is very different from the average; thus, the distance will be very large. The heatmap shows the probability that each pixel belongs to a tumor.

**Figure 6:**
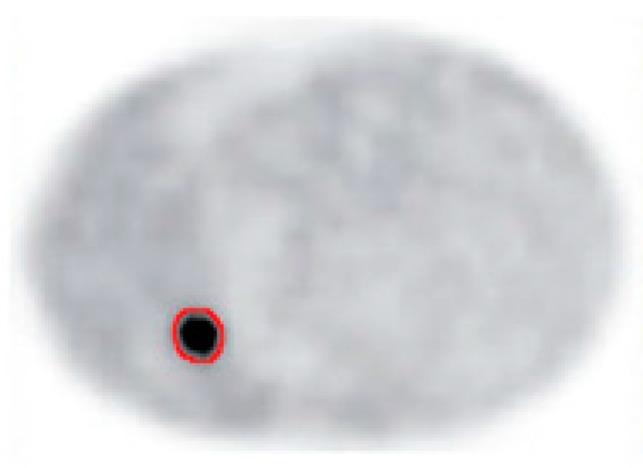
Output image with tumor contoured.

The final result of our segmentation algorithm for input image in Figure 2a is shown in the Figure 6. The tumor region is identified and delineated with good accuracy.

**Figure 7:**
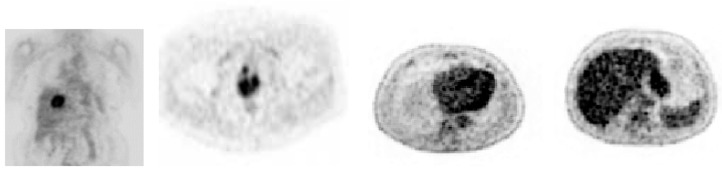

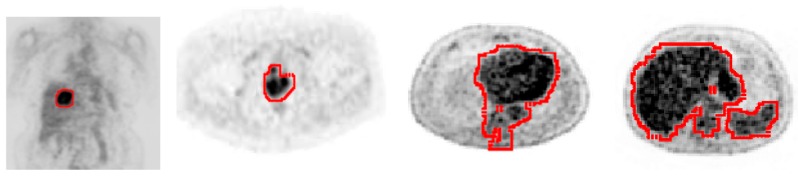
Sample input and output images.

Table 1 contains information about the size of the input image, the number of superpixels, size of extracted features (distance) after PCA and total execution time of some test images. The size of extracted features and number of superpixels are much smaller than the number of pixels of the image. Implementation was done in MATLAB 2016A (MathWorks, Natick, MA, United States) using core i5-4210U CPU, 1.7 GHz. The main concern of the paper was to design a fast PET tumor segmentation. As it can be seen from the table, the execution time of our proposed approach is very fast due to the following reasons: first, there is usually a small number of superpixels compared with pixels numbers in the image. This will save a lot of time in the algorithms after superpixels are extracted. Second, PCA again further reduces the dimension of data which is the input to clustering. In addition to that, MATLAB vectorization capability has also been extensively exploited throughout our implementation. Figure 7 shows sample input and output images. 

Our implementation can detect and contour tumors irrespective of their shape, location and number of tumors in the image. 

## 4. Conclusions

The main goal of this paper was to implement a fast algorithm to segment tumor in PET scan images. Detailed explanation of the mathematical formulation and discussion of intermediate and final results were also presented. To speed up execution time of the segmentation algorithm, PCA was applied on superpixels extracted from the contrast enhanced image. PCA was applied to reduce dimensionality and to study the internal statistics of superpixels. The segmentation was done using k-means clustering. Our implementation is able to localize and delineate the tumor with satisfactory accuracy and small execution time. 

The algorithm presented in this paper can be applied also in disciplines other than medicine to detect target areas that have distinctive internal statistics compared to the rest of the image. For a large sized image, the speed gained will be noticeable as a result of dimensionality reduction from superpixels and PCA.

Our algorithm has a promising result on PET scan images. Future work will aim at applying this analysis to CT and MRI images.

## Figures and Tables

**Table 1:** Size of images, superpixels, distance vector after Principal Component Analysis (PCA), and execution time.

	Size	No. Superpixels	Distance Vector Dimension	Execution Time (s)
Image 1	233 × 328	692	692	2.2
Image 2	233 × 328	500	500	2.4
Image 3	681 × 572	660	660	2.55
